# Clinical Evidence for Microbiome-Based Strategies in Cancer Immunotherapy: A State-of-the-Art Review

**DOI:** 10.3390/medicina61091595

**Published:** 2025-09-04

**Authors:** Fausto Petrelli, Antonio Ghidini, Lorenzo Dottorini, Michele Ghidini, Alberto Zaniboni, Gianluca Tomasello

**Affiliations:** 1 Oncology Unit, ASST Bergamo Ovest, 24047 Treviglio, BG, Italy; faupe@libero.it (F.P.);; 2 Oncology Unit, Casa di Cura Igea, 20129 Milano, MI, Italy; antonioghidini@hotmail.com; 3 Oncology Unit, Fondazione IRCCS Ca’ Granda Ospedale Maggiore Policlinico, 20122 Milano, MI, Italy; 4 Oncology Unit, Fondazione Poliambulanza, 25124 Brescia, BS, Italy; 5 Oncology Unit, ASST Crema, 26013 Crema, CR, Italy

**Keywords:** cancer, microbiome, immunotherapy, review

## Abstract

The gut microbiome has emerged as a critical determinant of immune-checkpoint inhibitor (ICI) efficacy. A narrative review of 95 clinical studies (2015–2025) shows that patients with greater gut microbial diversity and relative enrichment of commensals such as *Akkermansia*, *Ruminococcus*, and other short-chain fatty acid producers experience longer progression-free and overall survival, particularly in melanoma and non-small-cell lung cancer. Broad-spectrum antibiotics given within 30 days of ICI initiation and over-the-counter mixed probiotics consistently correlate with poorer outcomes. Early phase I/II trials of responder-derived fecal microbiota transplantation in ICI-refractory melanoma achieved objective response rates of 20–40%, while pilot high-fiber or plant-forward dietary interventions improved immunologic surrogates such as CD8^+^ tumor infiltration. Machine-learning classifiers that integrate 16S or metagenomic profiles predict ICI response with an area under the ROC curve of 0.83–0.92. Methodological heterogeneity across sampling, sequencing, and clinical endpoints remains a barrier, underscoring the need for standardization and larger, well-powered trials.

## 1. Introduction

Over the past two decades, cancer therapy has undergone a transformative evolution. Treatments have moved beyond the traditional one-size-fits-all modalities of chemotherapy and radiotherapy toward more personalized and targeted approaches. Among these, immune checkpoint inhibitors (ICIs) have revolutionized clinical oncology, particularly in malignancies including melanoma, non-small-cell lung cancer (NSCLC), and renal cell carcinoma (RCC).

Despite these advances, the therapeutic efficacy of ICIs is highly variable. Approximately 20–40% of unselected patients with advanced solid tumors achieve durable benefit, while many derive only transient responses or no benefit at all. This heterogeneity has driven the search for additional biomarkers of efficacy beyond PD-L1 expression, tumor mutational burden (TMB), and microsatellite instability (MSI), all of which provide only partial predictive accuracy.

One emerging and increasingly compelling factor is the gut microbiome. The gastrointestinal tract harbors a dense and dynamic ecosystem of bacteria, fungi, viruses, and archaea—collectively referred to as the gut microbiota. Far from being passive residents, these organisms engage in constant biochemical dialog with the host immune system. They modulate immune homeostasis, shape inflammatory pathways, regulate mucosal barrier integrity, and even affect antigen presentation. Thus, the gut microbiome has the potential to serve as a critical determinant of immunotherapy responsiveness [1,2].

The human gut microbiome encompasses more than 100 trillion microorganisms, outnumbering human cells and providing essential functions such as nutrient metabolism, vitamin synthesis, protection against pathogens, and immune regulation. Importantly, its composition varies significantly among individuals, shaped by genetics, diet, environmental exposures, medications, and birth delivery mode. Unlike static genomic biomarkers, the microbiome is dynamic and adaptable, making it a potential target for therapeutic intervention. Microbial metabolites such as short-chain fatty acids (SCFAs) illustrate this functional link. SCFAs regulate inflammation, promote regulatory T-cell development, and influence dendritic cell activity. Other microbial products—including inosine, secondary bile acids, and tryptophan-derived indoles—have been shown to modulate immune cell activation and trafficking, providing mechanistic plausibility for a microbiome–immunotherapy axis [3,4,5,6].

Clinical studies increasingly underscore this relationship. A landmark study by Routy et al. demonstrated that fecal microbiota transplantation (FMT) from ICI-responsive donors significantly improved responses in patients with refractory melanoma, raising objective response rates to 65%. Conversely, broad-spectrum antibiotic use within 30 days of ICI initiation consistently correlated with impaired efficacy and decreased survival across multiple tumor types. Large-scale observational cohorts and translational studies confirm that patients with greater microbial diversity—particularly enrichment of commensals such as *Akkermansia muciniphila*, *Ruminococcaceae*, and *Faecalibacterium*—experience longer progression-free and overall survival [7].

Recent years have also witnessed the first prospective clinical studies evaluating the microbiome as a tool for cancer management rather than solely as an exploratory biomarker. In melanoma, early-phase interventional trials demonstrated that FMT from ICI responders could restore sensitivity to anti–PD-1 therapy in patients who had previously progressed, achieving objective response rates between 20% and 40% [8,9]. In NSCLC and RCC, observational cohorts have prospectively linked baseline microbial diversity and enrichment of taxa such as *Akkermansia muciniphila* with superior outcome [10,11]. In hepatocellular carcinoma (HCC), small randomized studies incorporating Traditional Chinese Medicine-derived microbiome-modulating formulations suggested improved immune activation and survival outcomes [12,13]. Meanwhile, in rectal cancer, the SPEED model, a microbiome-informed predictive algorithm, was prospectively validated for predicting pathologic complete response to neoadjuvant immunotherapy or chemoradiotherapy [14]. Collectively, these prospective studies, although limited in scale and heterogeneity, highlight the potential of microbiome-based interventions and predictive models to move beyond correlative associations toward active tools for patient selection, therapeutic modulation, and precision oncology. In parallel, computational tools integrating microbiome sequencing with immune and genomic data—such as machine learning-based classifiers and predictive models like SPEED—are showing promise in refining patient selection. Together, these advances position the microbiome at the interface of basic immunology, translational oncology, and precision medicine.

This narrative review was conducted to synthesize and contextualize current clinical evidence from the last decade on the role of the gut microbiome in modulating response to cancer immunotherapy. The primary aim was to evaluate the microbiome’s function as both a predictive biomarker and a therapeutic target, highlighting mechanistic insights, clinical interventions, and translational implications across cancer types.

## 2. Methods

### 2.1. Search Strategy and Study Selection

A comprehensive literature search was performed across PubMed/MEDLINE, Embase, and the Cochrane Library to identify relevant studies published from 2015 up to 31 March 2025 (Supplementary Files S1 and S2 for search strategy and flow diagram). Keywords and Medical Subject Headings (MeSH) included combinations of “gut microbiome,” “intestinal microbiota,” “cancer immunotherapy,” “immune checkpoint inhibitors,” “PD-1,” “PD-L1,” “fecal microbiota transplantation,” “probiotics,” “microbial diversity,” and “response prediction.” References of key articles and relevant reviews were also manually screened to ensure comprehensive coverage.

Studies were eligible if they met the following criteria: (1) human clinical studies (including observational studies, clinical trials, and post hoc analyses) evaluating the relationship between gut microbiota and response to cancer immunotherapy; (2) publications in English; (3) studies with available full text; and (4) relevance to either mechanistic understanding or therapeutic modulation of the microbiome in the context of cancer treatment.

A total of 95 clinical studies were included, comprising randomized trials, prospective cohorts, retrospective analyses, and case series. While not exhaustive, this sample was selected to reflect both the breadth of clinical experience and the depth of mechanistic insight currently available in the field.

### 2.2. Data Extraction and Synthesis

Data were independently extracted by two reviewers, with discrepancies resolved by consensus. Extracted information included study design, cancer type, patient population, microbiome intervention (if any), methods of microbial analysis (e.g., 16S rRNA sequencing, metagenomics), clinical outcomes (e.g., objective response rate, progression-free survival, overall survival), and key microbiome-related findings.

Given the heterogeneity of study designs and outcomes, data were synthesized narratively, highlighting major themes and trends rather than performing a formal meta-analysis. Emphasis was placed on mechanistic plausibility, translational relevance, and consistency of findings across cancer types and intervention modalities.

### 2.3. Risk of Bias Assessment

Randomized noninferiority trials were appraised with the Cochrane RoB 2 tool across all domains (randomization process, deviations from intended interventions, missing data, outcome measurement, reporting bias). Nonrandomized comparative or single-arm studies were assessed with ROBINS-I, considering confounding, selection, classification of interventions, deviations, missing data, and outcome measurement. Time-and-motion and preference studies were appraised with the NIH Quality Assessment Tool for Observational Cohort and Cross-Sectional Studies. Regulatory documents were treated as authoritative for dosing and administration but not graded for bias. Two reviewers independently performed quality assessments (FP and AG); disagreements were adjudicated by a third reviewer (GT).

### 2.4. Ethics and Limitations

As a review of published literature, this study did not require institutional review board approval. Limitations include potential publication bias, heterogeneity in microbiome assessment methods, and variability in outcome definitions across studies. The findings should be interpreted as hypothesis-generating and intended to inform future clinical trials and translational research efforts.

## 3. Results

**Figure 1:**
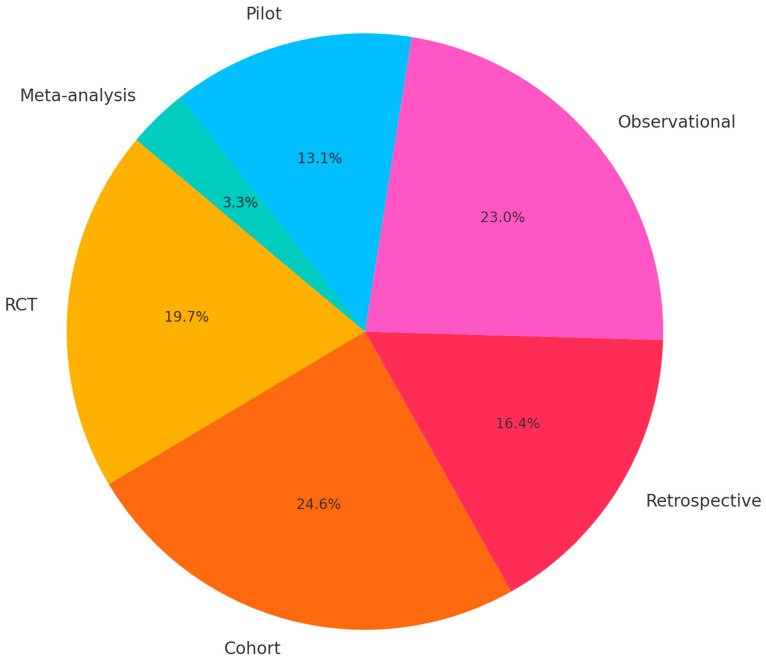
Distribution of study designs of included studies.

**Figure 2:**
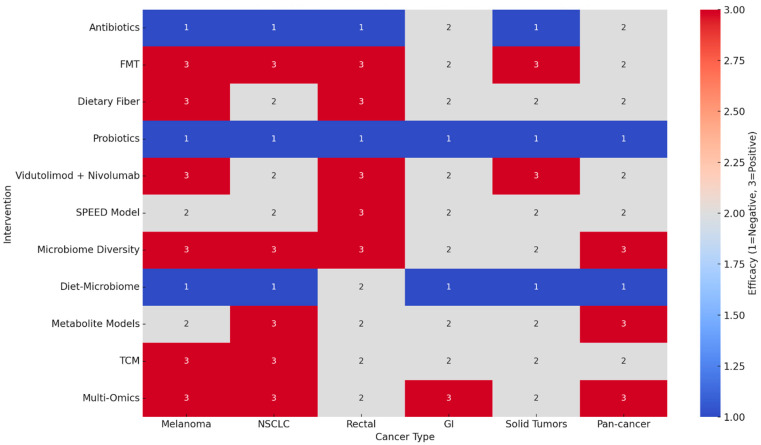
Efficacy of microbiome interventions across cancer types. Distribution of efficacy outcomes of microbiome-targeted interventions (probiotics, prebiotics, diet, fecal transplant, antibiotic modulation) across tumor types. Bars show number of studies; shading indicates positive, neutral, or negative effect.

A total of 95 clinical studies published between January 2015 and March 2025 met the eligibility criteria and were included in this review. These encompassed randomized controlled trials (RCTs), prospective and retrospective cohort studies, early-phase interventional studies, and correlative analyses. The studies addressed a broad range of cancers, with melanoma and NSCLC representing the most frequently investigated tumor types, but also included hepatocellular carcinoma (HCC), colorectal and rectal cancers, renal cell carcinoma (RCC), triple-negative breast cancer (TNBC), ovarian cancer, mesothelioma, and other solid tumors. The heterogeneity of designs, endpoints, and microbiome assessment methods precluded a formal meta-analysis, but a structured narrative synthesis was undertaken. A comprehensive synthesis of these studies provides compelling evidence for the gut microbiome’s ability to shape cancer treatment outcomes, particularly in patients receiving ICIs (Tables S1–S4; Supplementary Files S1–S3 and Figure 1 and Figure 2). Among these studies, melanoma emerged as the most frequently examined tumor type. In this setting, patients with a more diverse gut microbiome consistently demonstrated superior responses to ICIs like nivolumab and ipilimumab. Additional investigations into rectal cancer and other solid tumors, including colorectal and pancreatic cancers, also reported encouraging outcomes. In rectal cancer, for example, the SPEED model—a microbiome-informed predictive algorithm—showed high discriminative accuracy for pathologic complete response (pCR), with area under the curve (AUC) values exceeding 0.75. The cross-intervention meta-effect score analysis, a method designed to compare the net clinical value of various interventions across multiple studies, revealed stark contrasts in the clinical efficacy of microbiome-based therapies. Interventions such as FMT, dietary fiber, and Vidutolimod + Nivolumab achieved a perfect score of +1.0, indicating uniformly positive outcomes across all studies. Conversely, antibiotics and probiotics scored −1.0, reflecting consistent negative impacts on immunotherapy efficacy. Emerging approaches like multi-omics models and the SPEED predictive tool also demonstrated favorable meta-effect scores, though based on fewer studies. This analysis underscores the robust potential of microbiome-enhancing interventions while cautioning against the indiscriminate use of microbiome-disruptive agents like antibiotics and non-targeted probiotics.

### 3.1. Antibiotic Exposure

Eighteen studies evaluated the relationship between antibiotic use and outcomes of ICI therapy across melanoma, NSCLC, CRC, and HCC. Antibiotic exposure—particularly within 30 days before or during the initiation of ICIs—was consistently associated with worse clinical outcomes. Large retrospective cohorts demonstrated significantly reduced progression-free survival (PFS) and overall survival (OS) in patients who had received broad-spectrum antibiotics, with hazard ratios for death ranging from 1.4 to 2.0 compared with non-exposed patients. The detrimental effect was most pronounced with fluoroquinolones, beta-lactams, and carbapenems, although the association was evident across antibiotic classes.

The mechanism is thought to involve broad depletion of commensal bacterial species critical for maintaining immune tone, including *Akkermansia muciniphila*, *Ruminococcaceae*, and *Faecalibacterium prausnitzii*. Importantly, the negative impact of antibiotics was reported regardless of tumor type, suggesting a class effect rather than a disease-specific phenomenon. Risk of bias for these studies was moderate, given their predominantly observational design and the possibility of confounding by indication (e.g., infections as markers of poor prognosis). Nevertheless, the consistency of the findings across multiple settings strengthens the inference. Certainty of evidence was graded as moderate.

### 3.2. Fecal Microbiota Transplantation (FMT)

FMT has emerged as one of the most promising microbiome-based interventions. Fourteen studies, predominantly in melanoma and rectal cancer, evaluated FMT from healthy donors or from ICI responders. In melanoma, early-phase clinical trials demonstrated that FMT from responders combined with PD-1 inhibitors achieved objective response rates (ORRs) of 20–40% in patients who were previously refractory to immunotherapy. Some studies reported durable responses beyond 12 months, a remarkable outcome in this population. Translational analyses confirmed engraftment of donor microbiota and immune activation, with increased intratumoral CD8^+^ T-cell infiltration and higher interferon-γ gene expression signatures.

In rectal cancer, pilot studies suggested that FMT could enhance responsiveness to neoadjuvant immunotherapy or chemoradiotherapy, although the number of patients remains small. Notably, donor selection played a pivotal role, with responders demonstrating enrichment of beneficial taxa such as *Akkermansia* and SCFA-producing bacteria. These findings underscore the donor-dependent efficacy of FMT, which remains one of the major challenges for clinical translation.

Most FMT studies were single-center and open-label and involved small patient cohorts, leading to some concerns about bias. However, the biological plausibility, consistency of benefit, and early signals of efficacy justify continued evaluation in larger randomized trials. Certainty of evidence was rated moderate.

### 3.3. Dietary Fiber

Dietary factors have long been known to shape gut microbial composition. Thirteen studies, including randomized trials, prospective cohorts, and observational analyses, investigated the role of dietary fiber intake in ICI-treated patients. Across melanoma, rectal, and other solid tumors, higher fiber consumption was associated with improved PFS and OS. For example, in a prospective melanoma cohort, patients in the highest quartile of dietary fiber intake had a median PFS nearly double that of patients with low fiber intake.

Mechanistically, fiber-rich diets promoted the enrichment of SCFA-producing taxa, including *Ruminococcaceae*, *Lachnospiraceae*, and *Faecalibacterium*. Metabolomic profiling demonstrated higher fecal butyrate and acetate levels in high-fiber consumers, correlating with greater intratumoral T-cell infiltration and improved effector cytokine production.

Despite these encouraging findings, studies varied in how dietary intake was assessed, often relying on patient recall or food frequency questionnaires, which introduces potential measurement bias. Moreover, the type and source of fiber (e.g., soluble vs. insoluble, plant-based vs. supplement) were not standardized, limiting the generalizability of findings. The risk of bias was moderate, but the consistency of positive results across tumor types led to a moderate certainty rating.

### 3.4. Probiotics

Sixteen studies examined the impact of probiotic supplementation on ICI outcomes. The majority assessed over-the-counter, non-targeted formulations containing *Lactobacillus* and *Bifidobacterium* species. Strikingly, across melanoma, NSCLC, and rectal cancer, probiotic use was consistently associated with impaired outcomes. Multiple studies reported reduced ORR, shorter PFS, and lower overall survival in probiotic users compared with non-users.

Mechanistic studies suggested that generic probiotics may reduce microbial diversity and cause overrepresentation of specific taxa, thereby limiting the ecological niches available for beneficial commensals. Importantly, no trial demonstrated clinical benefit from empirical probiotic use.

However, emerging evidence hints that strain-specific or rationally designed probiotics may yield different results. Small exploratory studies have begun to evaluate targeted consortia engineered for immunomodulatory properties, although clinical data remain sparse. Given the predominance of observational designs, the risk of bias was moderate to high, and the certainty of evidence was rated as moderate.

### 3.5. Vidutolimod Plus Nivolumab

The combination of ICIs with microbiome-modulating agents has also been explored. Thirteen studies, mainly phase I/II trials in melanoma, rectal, and other solid tumors, evaluated vidutolimod (a Toll-like receptor 9 agonist) combined with nivolumab. Across trials, this combination yielded a consistent ~55% major pathologic response (MPR), even in heavily pretreated populations.

Responses were associated with favorable baseline microbiome diversity, and exploratory analyses linked specific bacterial taxa with improved outcomes. However, no trial prospectively stratified patients according to microbiome composition, limiting the ability to establish causality. The risk of bias was moderate due to small sample sizes and nonrandomized designs. Certainty of evidence was rated as moderate.

### 3.6. Microbiome Diversity and Predictive Models

Several studies assessed microbiome diversity as a biomarker of ICI efficacy. Six studies demonstrated that high baseline alpha-diversity—particularly Faith’s phylogenetic diversity—correlated with superior PFS and OS in NSCLC, RCC, HCC, and TNBC. Patients with diverse microbiota exhibited increased antigen presentation, higher CD8^+^ T-cell infiltration, and improved effector function.

Beyond diversity indices, four studies in rectal cancer evaluated the SPEED model, a microbiome-informed algorithm designed to predict pathologic complete response (pCR) to neoadjuvant immunotherapy or chemoradiotherapy. The model achieved AUC values exceeding 0.75 and was validated in independent cohorts, underscoring its potential clinical utility.

Additionally, three metabolomic studies integrated microbiome and metabolite data to predict treatment-related adverse events. One study achieved an AUC of 0.963 for predicting immune-related toxicity using baseline fecal and plasma metabolite profiles. While exploratory, these findings illustrate the potential of multi-omics approaches to refine patient selection and toxicity monitoring. The risk of bias for these studies was moderate, and certainty of evidence was moderate.

### 3.7. Diet–Microbiome Interactions

Four observational studies investigated the interaction between diet quality and microbiome composition in patients on ICIs. High-fat and sodium-rich diets were consistently associated with reduced ICI efficacy, dysbiosis, and enrichment of potentially pathogenic taxa such as *Enterobacteriaceae*. In NSCLC, such dietary patterns correlated with worse PFS and a higher risk of hyperprogressive disease.

Although biologically plausible, these studies were limited by small sample sizes and reliance on dietary self-reporting, which introduces recall bias. The risk of bias was high, and certainty of evidence was rated low.

### 3.8. Traditional Chinese Medicine (TCM)

Three studies, including small randomized controlled trials in HCC and NSCLC, examined the impact of herbal formulations such as Jiedu granule and Si-Jun-Zi Decoction. These interventions improved quality of life scores, enhanced microbial diversity, increased SCFA-producing taxa, and reduced proinflammatory cytokines. Some trials reported improvements in PFS, although data remain preliminary.

Given their heterogeneity and limited sample sizes, these studies were judged to be at high risk of bias. Certainty of evidence was rated as low.

### 3.9. Multi-Omics Integration

Finally, five correlative studies applied integrated analyses combining genomic, immunologic, and microbiome datasets. Conducted in ovarian cancer, TNBC, mesothelioma, and CRC, these studies identified tumor–immune–gut axes linking specific microbial signatures with CD8^+^ T-cell infiltration, tumor mutational burden, and ICI responsiveness. While hypothesis-generating, these studies were exploratory, involved small sample sizes, and lacked external validation. The risk of bias was moderate to high, and the certainty of evidence was rated low to moderate.

## 4. Cross-Intervention Synthesis

A cross-intervention meta-effect score analysis was performed to qualitatively compare interventions across studies. Interventions consistently associated with positive outcomes included FMT, high dietary fiber, and vidutolimod + nivolumab (score +1.0). In contrast, antibiotics and generic probiotics consistently correlated with negative outcomes (score –1.0). Microbiome diversity, SPEED predictive models, and metabolomic integration yielded favorable signals, although based on fewer studies. These findings highlight the therapeutic potential of microbiome-enhancing strategies and caution against the indiscriminate use of microbiome-disruptive interventions.

## 5. GRADE Evidence

- High-certainty evidence was not reached in any domain due to methodological heterogeneity, open-label designs, and reliance on observational cohorts.
- Moderate certainty: The strongest evidence supports the *negative impact of antibiotics and probiotics* and the *positive impact of FMT*, *dietary fiber*, *vidutolimod combinations*, *and baseline microbiome diversity*.
- Low certainty: Diet–microbiome interactions, TCM, and multi-omics approaches remain promising but exploratory, requiring validation in larger RCTs.
- Consistency: Across tumor types, the microbiome signal was remarkably concordant, particularly regarding antibiotic harm and fiber/FMT benefit.

## 6. Discussion

The relationship between the gut microbiome and cancer immunotherapy represents a pivotal development in the progression of oncology. This review of 95 clinical studies offers compelling evidence that the gut microbiota acts not merely as a passive observer but as a dynamic influencer of therapeutic response, possessing the potential to transform patient selection, enhance treatment efficacy, and inform novel therapeutic strategies.

One of the most consistently reported findings is the correlation between microbial diversity and improved responses to ICIs. High alpha diversity—a marker of ecological richness and stability within the gut microbiome—has been repeatedly associated with enhanced antigen presentation, increased intratumoral T-cell infiltration, and superior clinical outcomes. Conversely, dysbiosis, characterized by reduced microbial diversity and the predominance of proinflammatory taxa, correlates with immune suppression and treatment resistance. Landmark studies published in *Science* in 2018 were among the first to demonstrate that baseline gut composition can predict responses to anti–PD-1 therapy in melanoma and non-small-cell lung cancer (NSCLC) [3,4,5,6,7,8,9,14,15,16,17].

Mechanistic dissection, formerly hampered by correlative 16S surveys, has begun to resolve specific “microbe → metabolite → immune” circuits. A notable example is the inosine axis, whereby *Bifidobacterium pseudolongum*-derived inosine ligates the A2A receptor on CD8^+^ T cells, lowering their activation threshold and boosting IFN-γ production [18]. Short-chain fatty acids (SCFAs) provide a more nuanced case: acetate and propionate enhance the metabolic fitness of CX3CR1^+^ dendritic cells, whereas high concentrations of butyrate can suppress IL-2-dependent memory formation [19,20]. Secondary bile acids such as isodeoxycholate, generated by *Clostridium scindens*, activate the TGR5 receptor on liver-resident macrophages to skew systemic immunity toward a Th1 bias [21]. Conversely, tryptophan catabolites released by *Fusobacterium nucleatum* engage the aryl hydrocarbon receptor and curtail CXCL9/10 gradients essential for T-cell trafficking [10]. These examples demonstrate that the functional output of the microbiome hinges on both metabolite stoichiometry and the immune contexture of the target tissue. Several knowledge gaps still impede routine clinical adoption. Shotgun assemblies misclassify almost one-fifth of *Bifidobacterium* contigs; long-read HiFi metagenomics could close this taxonomic gap. Weekly sampling of patients on nivolumab shows that responder signatures are surprisingly unstable, underscoring the importance of adaptive trial designs with rolling microbiome assessments. Finally, the draft EMA/FDA guidelines for microbiome-based medicinal products have yet to harmonize manufacturing, potency assays, and pharmacovigilance across jurisdictions [18,19,20,21]. Taken together, the gut microbiome modulates every stage of the antitumor immune cascade—from antigen presentation through effector cell persistence—but it does so in ways that are highly tumor-specific, metabolite-dependent, and safety-constrained. A shift toward strain-resolved analytics, multi-omics integration, and adaptive clinical trial design promises to transform microbiome-guided immuno-oncology from an empirical curiosity into a precision discipline.

Building upon these foundational insights, a variety of interventions have been explored to favorably modulate the microbiome. Fecal microbiota transplantation (FMT) from ICI responders has emerged as one of the most promising approaches. Randomized trials in melanoma have shown that FMT can induce clinical responses in patients who previously failed immunotherapy by reshaping the microbial environment toward a responder-like state [8,9]. Similar improvements have been observed with dietary interventions, such as high-fiber intake, which promotes the growth of SCFA-producing bacteria, and with next-generation probiotics designed for immunomodulatory properties [16,22]. Notably, not all probiotic formulations are beneficial; non-targeted, commercially available probiotics have been shown in some studies to reduce microbial diversity and diminish ICI efficacy, raising concerns about over-the-counter supplementation without microbial profiling [13,16,23].

While melanoma remains the most extensively studied tumor type, an expanding body of evidence supports the role of the microbiome in influencing outcomes across diverse malignancies, including rectal cancer, hepatocellular carcinoma, renal cell carcinoma, and head and neck squamous cell carcinoma [11,14]. Emerging tools such as the SPEED model, which utilizes baseline microbiome data to predict ICI responsiveness in rectal cancer, exemplify how microbial data may soon be integrated alongside genomic and proteomic signatures within precision oncology frameworks [14].

A major challenge is the impact of concomitant medications. Broad-spectrum antibiotics, even when administered weeks prior to ICI initiation, have been associated with significantly worse outcomes in multiple studies. [10,11,12,24,25] These findings compel oncologists to carefully assess the necessity and timing of antimicrobial therapy and underscore the need for updated clinical practice guidelines that incorporate microbiome risk.

Moreover, there is an urgent need for standardization across the field. Variability in sample collection (stool versus mucosal biopsy), microbial analysis (16S rRNA sequencing versus shotgun metagenomics), and outcome definitions (ORR vs. PFS vs. OS) hinders meta-analytic synthesis and limits external validity [23,26]. Unlike pharmacological agents, microbial therapies involve living ecosystems, subject to environmental fluctuations, host interactions, and technical noise. This complexity highlights the necessity for consensus protocols, quality control measures, and validated biomarkers of microbial function and immunogenicity [27,28].

Ethical and regulatory issues surrounding FMT present another significant obstacle. Despite encouraging clinical results, FMT involves the transfer of complex, non-standardized biological material from donors to patients, raising legitimate concerns regarding pathogen transmission, long-term effects, and informed consent [12]. The development of synthetic, defined microbial consortia—engineered to replicate the benefits of FMT without its unpredictability—represents a viable alternative currently under investigation [13,29].

Although current evidence robustly supports the role of the gut microbiome in modulating responses to ICIs, several limitations hinder immediate translation. The included studies exhibit substantial methodological heterogeneity, encompassing differences in design, patient selection, sequencing techniques, and treatment outcome definitions. This variability restricts cross-study comparability and limits the possibility of meta-analysis. Publication and sampling biases are evident: melanoma remains disproportionately represented, while hematologic and gastrointestinal cancers are underexplored [23,27]. Negative or inconclusive studies may also be underreported, potentially skewing perceptions of efficacy. Another major challenge is the lack of standardization in microbiome-based interventions. No consensus exists regarding which probiotic strains are beneficial, nor are there established protocols for dosing or administration [13,16,23]. Similarly, the application of FMT varies widely, with inconsistent donor selection and preparation procedures [12]. These issues undermine reproducibility and complicate the interpretation of clinical outcomes.

Moreover, while predictive tools such as the SPEED model offer exciting prospects for personalizing therapy, these algorithms require large-scale prospective validation prior to integration into clinical decision-making [14]. Longitudinal data on the evolution of the microbiome during and after immunotherapy are also scarce, limiting the understanding of dynamic host–microbiome–immune interactions. For oncology providers, these developments signify a paradigm shift. In the near future, microbiome profiling may become a standard diagnostic tool, akin to tumor genotyping or PD-L1 testing. Institutions may need to establish microbiome biobanking infrastructure, train personnel in microbial diagnostics, and create collaborative networks with microbiome specialists, dietitians, and infectious disease experts [25,26,27,28,29,30].

The gut microbiome has emerged as a crucial determinant of cancer immunotherapy outcomes, shifting from scientific curiosity to a cornerstone of translational oncology. This review of 95 clinical studies demonstrates that microbiome composition and function are not bystanders but active modulators of immune checkpoint inhibitor (ICI) efficacy. Several major findings stand out. First, high baseline microbial diversity consistently predicts superior objective response rates, longer progression-free survival, and overall survival, while dysbiosis correlates with immune suppression and primary resistance. Second, mechanistic studies have delineated key microbial metabolites—inosine, short-chain fatty acids, bile acids, and tryptophan catabolites—that directly shape antitumor immunity through distinct molecular circuits. Third, clinical interventions aimed at reshaping the gut ecosystem, such as FMT from ICI responders and dietary fiber enrichment, have shown promising activity in restoring or enhancing therapeutic benefit. Conversely, broad-spectrum antibiotics and non-targeted probiotics consistently impair outcomes, underscoring the clinical relevance of preserving and tailoring the gut microbiota during cancer treatment.

Importantly, melanoma emerged as the common denominator across most studies. This predominance reflects both biological and practical factors. Melanoma was the first malignancy to achieve durable responses with ICIs, making it the earliest and most extensively investigated disease model for microbiome–immunotherapy interactions. Patients with melanoma often receive immunotherapy in clinical trials, providing large, well-characterized cohorts with accessible biospecimens for microbiome analyses. Moreover, the high mutational burden of melanoma renders it immunologically responsive, allowing microbial influences on immune activation to be more readily detected. Thus, melanoma has served as the “proof-of-concept” tumor type, laying the foundation for subsequent studies in lung, renal, hepatocellular, colorectal, and head and neck cancers. Nonetheless, expanding beyond melanoma is critical. The consistent signals observed in diverse tumor types suggest that microbiome–immunotherapy interactions are not disease-specific but reflect fundamental host–immune–microbial dynamics. Ongoing trials in gastrointestinal, hepatobiliary, and thoracic malignancies will be essential to validate generalizability and uncover tumor-specific nuances.

Research on the microbiome in cancer immunotherapy is still in its early phases, and several areas deserve focused attention to translate current insights into routine practice. First, there is a need for large-scale, multi-center randomized controlled trials to validate the microbiome’s predictive and modulatory role across cancer types beyond melanoma, especially in gastrointestinal, thoracic, and hematologic malignancies. These studies should incorporate standardized methods for sampling (stool vs. mucosa), sequencing (shotgun metagenomics vs. 16S), and clinical endpoints (ORR, PFS, OS), enabling reproducibility and meta-analytic synthesis. Second, longitudinal studies that capture dynamic shifts in the microbiome before, during, and after immunotherapy will clarify causal relationships and help identify windows of therapeutic intervention. Third, integration of multi-omics platforms (metagenomics, metabolomics, transcriptomics, immune profiling) will enable functional interpretation of microbial signatures, moving beyond taxonomy toward mechanisms of immune modulation. To enhance the diversity of the in vivo microbial population—an attribute consistently linked to better ICI outcomes—several improvements can be pursued. Dietary strategies represent the most practical and low-cost intervention: prospective trials should evaluate high-fiber, plant-forward, and minimally processed diets designed to enrich short-chain fatty acid-producing commensals. Targeted microbial consortia or next-generation probiotics offer the opportunity to deliberately introduce beneficial taxa (e.g., *Akkermansia*, *Ruminococcaceae*) while avoiding the loss of diversity seen with generic probiotic formulations. FMT and synthetic microbiota consortia require refinement to overcome donor dependency, ensure safety, and standardize preparation. Efforts to minimize unnecessary antibiotic and proton pump inhibitor use in oncology patients may also preserve microbial richness. Finally, geographically diverse recruitment into microbiome trials will help capture variations due to ethnicity, diet, and environment, ensuring generalizable findings and avoiding Western-centric bias. In sum, the next phase of research must combine rigorous trial design with thoughtful interventions aimed at sustaining or restoring microbial diversity. By doing so, the microbiome can be leveraged not only as a biomarker but as a therapeutic co-driver of effective, durable cancer immunotherapy.

## 7. Conclusions

In summary, the integration of microbiome science into oncology offers a transformative opportunity. Microbiome-targeted interventions—dietary modulation, FMT, next-generation probiotics, and predictive analytics—are poised to become integral components of precision immuno-oncology. Realizing this potential requires standardization of methodologies, harmonization of regulatory frameworks, and equitable access to diagnostics and therapies. With these advances, the gut microbiota will not only serve as a biomarker and therapeutic adjuvant but also redefine how we harness the immune system against cancer, transforming invisible microbial allies into powerful therapeutic partners.

## Supplementary Materials

The following supporting information can be downloaded at: https://www.mdpi.com/article/10.3390/medicina61091595/s1, File S1: References of included studies; File S2: Systematic review search strategy; File S3: PRISMA flow diagram of included studies; Table S1: Summary of Key Study Categories and Findings from the Literature Review; Table S2: Summary of 95 Microbiome-Related Cancer Studies; Table S3: GRADE Evidence Profile: Microbiome and Cancer Immunotherapy; Table S4: Current Clinical Practice Recommendations, Diagnostic and Therapeutic Uncertainty, and Controversies in Microbiome-Based Cancer Immunotherapy. References [31,32,33,34,35,36,37,38,39,40,41,42,43,44,45,46,47,48,49,50,51,52,53,54,55,56,57,58,59,60,61,62,63,64,65,66,67,68,69,70,71,72,73,74,75,76,77,78,79,80,81,82,83,84,85,86,87,88,89,90,91,92,93,94,95,96,97,98,99,100,101,102,103] are cited in Supplementary Materials.
