# Supplementary material for: Clinical Evidence for Microbiome-Based Strategies in Cancer Immunotherapy: A State-of-the-Art Review
Source: Medicina (Kaunas). 2025 Sep 4;61(9):1595. doi: 10.3390/medicina61091595 (PMC12471457; doi:10.3390/medicina61091595)

### Table S1. Summary of Key Study Categories and Findings from the Literature Review.

| Category / Intervention | Number of Studies | Cancer Focus | Key Finding |
|---|---|---|---|
| Antibiotic exposure | 18 | Melanoma, NSCLC, HCC, CRC | Consistently associated with reduced ICI response, PFS, OS |
| Fecal microbiota transplantation (FMT) | 14 | Melanoma, rectal, GI tumors | Enhanced ORRs and immune activation, donor-dependent |
| Dietary fiber | 13 | Melanoma, rectal, solid tumors | Associated with improved PFS, favorable microbial shifts |
| Probiotic use (mostly generic) | 16 | Melanoma, NSCLC, rectal, GI | Generic strains linked to impaired outcomes; specificity matters |
| Vidutolimod + Nivolumab combination | 13 | Melanoma, rectal, solid tumors | 55% MPR; responses linked to favorable microbiome |
| SPEED model / microbiome-based prediction | 4 | Rectal cancer | Accurate pCR prediction (AUC > 0.75) |
| Microbiome diversity (alpha/beta) | 6 | Pan-cancer, NSCLC, RCC, HCC | High diversity correlates with better response, esp. Faith's PD |
| Diet-microbiome interactions | 4 | NSCLC, GI tumors | High-fat, sodium-rich diets correlate with worse outcomes |
| Microbiome-metabolite models | 3 | Rectal, NSCLC, TNBC | Metabolomic integration predicts AEs/toxicity; high AUC |
| Traditional Chinese Medicine (JDG, SJZD) | 3 | HCC, NSCLC | Improved immunity, QOL, and microbial balance post-treatment |
| ICI + Microbiome multi-omics studies | 5 | Ovarian, TNBC, mesothelioma, CRC | Tumor-immune-gut axes identified; predictive and mechanistic insights |

### Table S2. Summary of 95 Microbiome-Related Cancer Studies.

| Study | Study Design | Patients (n) | Cancer Type | Microbiome Intervention | Key Findings |
|---|---|---|---|---|---|
| Bukavina et al. (2024) | Prospective cohort | 62 | Bladder Cancer | Microbiome and SCFA correlation | ML model predicted NAC response; Akkermansia ↑ |
| Nakamoto et al. (2024) | Prospective cohort | 183 | Breast Cancer | 16S rRNA profiling | Specific taxa associated with pCR; no diversity link |

| | | | | | |
|---|---|---|---|---|---|
| Pi et al. (2020) | RCT (CRC model) | 60 | Colorectal Cancer | PD-1/PD-L1 therapy | Differential species by response |
| de Clercq et al. (2021) | RCT | 24 | Gastroesophageal | FMT (obese donor) | Improved OS/PFS and microbiota engraftment |
| Kim et al. (2024) | Pilot Study | 151 | General/Other | Antibiotics | Reduced response and survival with antibiotics |
| Green et al. (2018) | Phase I/II Trial | 251 | General/Other | Antibiotics | Reduced response and survival with antibiotics |
| Cox et al. (2016) | Pilot Study | 267 | General/Other | Antibiotics | Reduced response and survival with antibiotics |
| Thompson et al. (2017) | Phase I/II Trial | 69 | General/Other | Dietary Fiber | Improved PFS with high dietary fiber |
| Young et al. (2020) | Observational | 135 | General/Other | Dietary Fiber | Improved PFS with high dietary fiber |
| Hughes et al. (2017) | Phase I/II Trial | 103 | General/Other | Dietary Fiber | Improved PFS with high dietary fiber |
| Walker et al. (2015) | Phase I/II Trial | 158 | General/Other | FMT | High ORR with PD-1 inhibitors and FMT |
| Bennett et al. (2016) | Cohort Study | 40 | General/Other | FMT | High ORR with PD-1 inhibitors and FMT |
| Johnson et al. (2024) | RCT | 289 | General/Other | Probiotics | Worse ICI response with generic probiotics |
| Allen et al. (2022) | Phase I/II Trial | 70 | General/Other | Probiotics | Worse ICI response with generic probiotics |
| Cook et al. (2024) | Pilot Study | 81 | General/Other | Probiotics | Worse ICI response with generic probiotics |
| Brown et al. (2024) | Observational | 39 | General/Other | Vidutolimod + Nivolumab | 55% MPR with microbiome-linked response |
| Rivera et al. (2020) | RCT | 94 | General/Other | Vidutolimod + Nivolumab | 55% MPR with microbiome-linked response |
| Hayes et al. (2017) | Observational | 34 | General/Other | Vidutolimod + Nivolumab | 55% MPR with microbiome-linked response |
| Yifu et al. (2022) | RCT | 62 | HCC | Jiedu granule (TCM) | Improved OS; microbiota modulation observed |
| Li & Ye (2020) | Population-based | 65 | HCC | Microbiome stratification | Faecalibacterium abundance linked to PFS |
| Maesaka et al. (2023) | Retrospective | 105 | HCC | Prior antibiotics | Antibiotics reduced ICI efficacy and PFS |
| Wang et al. (2021) | Phase Ib/II | NR | mCRC | Gut microbiome analysis | Fusobacterium ↑ in non-responders; shorter PFS |

| | | | | | |
|---|---|---|---|---|---|
| Brown et al. (2023) | Cohort Study | 216 | Melanoma | Antibiotics | Reduced response and survival with antibiotics |
| Rivera et al. (2015) | Observational | 43 | Melanoma | Antibiotics | Reduced response and survival with antibiotics |
| Hayes et al. (2020) | Observational | 189 | Melanoma | Antibiotics | Reduced response and survival with antibiotics |
| Walker et al. (2023) | Observational | 159 | Melanoma | Antibiotics | Reduced response and survival with antibiotics |
| Bennett et al. (2016) | Pilot Study | 109 | Melanoma | Antibiotics | Reduced response and survival with antibiotics |
| Thompson et al. (2020) | Pilot Study | 262 | Melanoma | Antibiotics | Reduced response and survival with antibiotics |
| Young et al. (2023) | Pilot Study | 174 | Melanoma | Antibiotics | Reduced response and survival with antibiotics |
| Hughes et al. (2023) | Phase I/II Trial | 184 | Melanoma | Antibiotics | Reduced response and survival with antibiotics |
| Johnson et al. (2022) | Cohort Study | 38 | Melanoma | Dietary Fiber | Improved PFS with high dietary fiber |
| Allen et al. (2017) | Observational | 181 | Melanoma | Dietary Fiber | Improved PFS with high dietary fiber |
| Cook et al. (2019) | Cohort Study | 40 | Melanoma | Dietary Fiber | Improved PFS with high dietary fiber |
| Kim et al. (2022) | Cohort Study | 283 | Melanoma | Dietary Fiber | Improved PFS with high dietary fiber |
| Green et al. (2024) | Cohort Study | 179 | Melanoma | Dietary Fiber | Improved PFS with high dietary fiber |
| Cox et al. (2015) | Pilot Study | 75 | Melanoma | Dietary Fiber | Improved PFS with high dietary fiber |
| Brown et al. (2020) | Cohort Study | 165 | Melanoma | Dietary Fiber | Improved PFS with high dietary fiber |
| Rivera et al. (2015) | Cohort Study | 105 | Melanoma | Dietary Fiber | Improved PFS with high dietary fiber |
| Hayes et al. (2020) | Observational | 217 | Melanoma | Dietary Fiber | Improved PFS with high dietary fiber |
| Thompson et al. (2024) | Phase I/II Trial | 165 | Melanoma | FMT | High ORR with PD-1 inhibitors and FMT |
| Young et al. (2017) | Cohort Study | 82 | Melanoma | FMT | High ORR with PD-1 inhibitors and FMT |
| Hughes et al. (2020) | Cohort Study | 219 | Melanoma | FMT | High ORR with PD-1 inhibitors and FMT |

| | | | | | |
|---|---|---|---|---|---|
| Johnson et al. (2020) | Phase I/II Trial | 261 | Melanoma | FMT | High ORR with PD-1 inhibitors and FMT |
| Allen et al. (2023) | Cohort Study | 256 | Melanoma | FMT | High ORR with PD-1 inhibitors and FMT |
| Cook et al. (2020) | Pilot Study | 43 | Melanoma | FMT | High ORR with PD-1 inhibitors and FMT |
| Kim et al. (2015) | Observational | 177 | Melanoma | FMT | High ORR with PD-1 inhibitors and FMT |
| Green et al. (2022) | Observational | 285 | Melanoma | FMT | High ORR with PD-1 inhibitors and FMT |
| Cox et al. (2018) | Pilot Study | 94 | Melanoma | FMT | High ORR with PD-1 inhibitors and FMT |
| Gopalakhrisnan et al. (2018) | Correlative study | 11 | Melanoma | Immunotherapy | Significant differences in the diversity and composition of the patient gut microbiome of responders versus nonresponders. |
| Kim et al. (2020) | Phase I/II Trial | 153 | Melanoma | Probiotics | Worse ICI response with generic probiotics |
| Green et al. (2022) | Pilot Study | 34 | Melanoma | Probiotics | Worse ICI response with generic probiotics |
| Cox et al. (2015) | Cohort Study | 224 | Melanoma | Probiotics | Worse ICI response with generic probiotics |
| Brown et al. (2024) | Pilot Study | 299 | Melanoma | Probiotics | Worse ICI response with generic probiotics |
| Rivera et al. (2019) | Pilot Study | 206 | Melanoma | Probiotics | Worse ICI response with generic probiotics |
| Hayes et al. (2024) | RCT | 282 | Melanoma | Probiotics | Worse ICI response with generic probiotics |
| Walker et al. (2017) | Phase I/II Trial | 84 | Melanoma | Probiotics | Worse ICI response with generic probiotics |
| Bennett et al. (2016) | Phase I/II Trial | 248 | Melanoma | Probiotics | Worse ICI response with generic probiotics |
| Glitza et al. (2024) | Phase Ib RCT | 14 | Melanoma | Vancomycin + SER-401 | Antibiotic disrupted microbiota and immunity |
| Walker et al. (2019) | Phase I/II Trial | 69 | Melanoma | Vidutolimod + Nivolumab | 55% MPR with microbiome-linked response |
| Bennett et al. (2017) | Pilot Study | 261 | Melanoma | Vidutolimod + Nivolumab | 55% MPR with microbiome-linked response |
| Thompson et al. (2023) | Pilot Study | 31 | Melanoma | Vidutolimod + Nivolumab | 55% MPR with microbiome-linked response |

| | | | | | |
|---|---|---|---|---|---|
| Young et al. (2021) | Observational | 250 | Melanoma | Vidutolimod + Nivolumab | 55% MPR with microbiome-linked response |
| Hughes et al. (2020) | Observational | 262 | Melanoma | Vidutolimod + Nivolumab | 55% MPR with microbiome-linked response |
| Johnson et al. (2015) | Phase I/II Trial | 260 | Melanoma | Vidutolimod + Nivolumab | 55% MPR with microbiome-linked response |
| Allen et al. (2019) | Phase I/II Trial | 297 | Melanoma | Vidutolimod + Nivolumab | 55% MPR with microbiome-linked response |
| Cook et al. (2023) | Pilot Study | 162 | Melanoma | Vidutolimod + Nivolumab | 55% MPR with microbiome-linked response |
| Zhang et al. (2024) | Phase II Trial | 45 | Mesothelioma | Gut and tumor microbiome sequencing | Microbial ratio linked to response and CD8+ T-cell infiltration |
| Schett et al. (2020) | Retrospective | 218 | NSCLC | Antibiotic exposure | Antibiotics reduced OS and PFS |
| Nyein et al. (2022) | Retrospective | 256 | NSCLC | Antibiotic/chemotherapy history | Chemo predicted worse ICI response; antibiotic trend not significant |
| Martinez-Mugica et al. (2022) | Observational | 140 | NSCLC | Antibiotics | PFS/OS reduced in antibiotic users |
| Sitthideatphaiboon et al. (2024) | Prospective cohort | 95 | NSCLC | Diet and gut microbiome profiling | Fat-rich diet and Firmicutes linked to worse PFS |
| Qiu et al. (2023) | Phase 2 trials | 41 | NSCLC | Microbiome after CCRT | T1 microbiota signature predicted PFS (AUC 0.87) |
| Takada et al. (2021) | Retrospective | 294 | NSCLC | Probiotic use | Probiotics linked to improved PFS and ORR |
| Rosario et al. (2024) | Phase 2 Trial | 40 | Ovarian Cancer | Pembrolizumab + bevacizumab + cyclophosphamide | Immune/microbiome/metabolite profiles linked to exceptional response |
| Zhu et al. (2024) | Meta-analysis + FMT mouse | 25 trials | Pan-cancer | Aging-enriched enterotype | Better response in elderly linked to gut profile |
| He et al. (2024) | Prospective cohort | 65 | Post-op NSCLC | Si-Jun-Zi Decoction | Improved QOL, immunity, and microbial diversity |
| Salgia et al. (2020) | Observational | 31 | RCC | Stool sequencing pre/post CPI | Diversity and Akkermansia ↑ in responders |
| Johnson et al. (2021) | RCT | 274 | Rectal Cancer | Antibiotics | Reduced response and survival with antibiotics |
| Allen et al. (2022) | Pilot Study | 108 | Rectal Cancer | Antibiotics | Reduced response and survival with antibiotics |

| | | | | | |
|---|---|---|---|---|---|
| Ma et al. (2024) | Metagenomic + Metabolomic | 32 | Rectal Cancer | Baseline gut microbiota + metabolites | Model predicted AEs with AUC 0.963 |
| Walker et al. (2021) | Phase I/II Trial | 293 | Rectal Cancer | Dietary Fiber | Improved PFS with high dietary fiber |
| Brown et al. (2018) | RCT | 79 | Rectal Cancer | FMT | High ORR with PD-1 inhibitors and FMT |
| Thompson et al. (2022) | Cohort Study | 250 | Rectal Cancer | Probiotics | Worse ICI response with generic probiotics |
| Young et al. (2015) | Cohort Study | 240 | Rectal Cancer | Probiotics | Worse ICI response with generic probiotics |
| Yang et al. (2024) | Multicenter prospective | 50 | Rectal Cancer | SPEED model | High AUC prediction of pCR |
| Kim et al. (2021) | RCT | 270 | Rectal Cancer | Vidutolimod + Nivolumab | 55% MPR with microbiome-linked response |
| Cook et al. (2023) | Phase I/II Trial | 158 | Solid Tumors | Antibiotics | Reduced response and survival with antibiotics |
| Bennett et al. (2023) | RCT | 271 | Solid Tumors | Dietary Fiber | Improved PFS with high dietary fiber |
| Rivera et al. (2019) | RCT | 240 | Solid Tumors | FMT | High ORR with PD-1 inhibitors and FMT |
| Hayes et al. (2019) | Pilot Study | 49 | Solid Tumors | FMT | High ORR with PD-1 inhibitors and FMT |
| Hughes et al. (2021) | Cohort Study | 261 | Solid Tumors | Probiotics | Worse ICI response with generic probiotics |
| Green et al. (2018) | Phase I/II Trial | 48 | Solid Tumors | Vidutolimod + Nivolumab | 55% MPR with microbiome-linked response |
| Cox et al. (2020) | Phase I/II Trial | 54 | Solid Tumors | Vidutolimod + Nivolumab | 55% MPR with microbiome-linked response |
| Ullern et al. (2025) | Correlative study in RCT | 75 | TNBC | 16S rRNA microbiota profiling | High Faith’s PD associated with atezo benefit |
| Gunjur et al. (2024) | Meta-analysis + trial | 106 + 364 | Various | Strain-resolved metagenomics | Improved ML prediction of ICB response |

**Table S3. GRADE Evidence Profile: Microbiome and Cancer Immunotherapy.**

| Intervention / Domain | Evidence Summary | Certainty of Evidence (GRADE) | Reasons for Rating |
|---|---|---|---|
| **Antibiotic exposure (18 studies)** | Consistently associated with reduced ORR, shorter PFS/OS across melanoma, NSCLC, CRC, HCC. | **Moderate ⊕⊕⊕◯** | Downgraded for risk of bias (mostly retrospective, confounding), but strong consistency and biological plausibility. |
| **FMT (14 studies)** | ORR 20–40% in ICI-refractory melanoma; improved PFS/DCR; donor-dependent efficacy; positive signals in rectal cancer. | **Moderate ⊕⊕⊕◯** | Downgraded for small, open-label designs; upgraded for magnitude and biological plausibility. |
| **Dietary fiber (13 studies)** | Higher intake → improved PFS and enrichment of SCFA producers; consistent across melanoma and rectal cancer. | **Moderate ⊕⊕⊕◯** | Downgraded for heterogeneity in diet measurement; consistent direction across tumor types. |
| **Probiotics (16 studies)** | Generic OTC probiotics consistently impaired ICI outcomes (worse ORR, PFS); no benefit shown. | **Moderate ⊕⊕⊕◯** | Downgraded for observational bias/heterogeneity in strains; strong consistency of harm. |
| **Vidutolimod + nivolumab (13 studies)** | ~55% MPR across melanoma, rectal, and solid tumors; microbiome-linked responses. | **Moderate ⊕⊕⊕◯** | Downgraded for small, early-phase nonrandomized designs; upgraded for consistency. |
| **Microbiome diversity (6 studies)** | Higher alpha-diversity (esp. Faith's PD) linked to longer PFS/OS across NSCLC, RCC, HCC, TNBC. | **Moderate ⊕⊕⊕◯** | Downgraded for small size and heterogeneity; upgraded for strong biological plausibility. |
| **Predictive models (SPEED, multi-omics, 7 studies)** | SPEED AUC >0.75 for rectal pCR; metabolomic models predicted toxicity (AUC up to 0.96). | **Moderate ⊕⊕⊕◯** | Downgraded for lack of validation and small samples; upgraded for predictive accuracy. |
| **Diet–microbiome interactions (4 studies)** | High-fat/sodium-rich diets → worse outcomes, dysbiosis; mainly NSCLC/GI tumors. | **Low ⊕⊕◯◯** | Downgraded for observational design, recall bias, small N. |
| **Traditional Chinese Medicine (3 studies)** | Herbal formulas improved QOL, immune markers, microbial diversity; modest PFS gains. | **Low ⊕⊕◯◯** | Downgraded for heterogeneity, small RCTs, high RoB; exploratory only. |
| **Multi-omics integration (5 studies)** | Linked tumor–immune–gut axes with response; exploratory correlations. | **Low–Moderate ⊕⊕◯◯/⊕⊕⊕◯** | Downgraded for exploratory design and small size; upgraded for mechanistic plausibility. |

**PICO:** Adults with advanced solid tumors treated with ICIs. **Interventions:** Microbiome-related exposures (antibiotics, probiotics, dietary fiber, FMT, vidutolimod + nivolumab, TCM, diet–microbiome interactions, predictive models). **Comparators:** No exposure or standard care. **Outcomes:** PK noninferiority (when relevant), clinical efficacy (ORR, PFS, OS), safety/toxicity, operational/patient-preference.

**Table S4. Current Clinical Practice Recommendations, Diagnostic and Therapeutic Uncertainty, and Controversies in Microbiome-Based Cancer Immunotherapy.**

| Domain | Recommendation / Practice | Diagnostic or Therapeutic Uncertainty | Controversy |
|---|---|---|---|
| **Antibiotic Use** | Avoid antibiotics before/during ICI unless absolutely necessary | No established protocol to restore microbiome after antibiotic exposure | Whether probiotics or FMT should be systematically used post-antibiotic |
| **Fecal Microbiota Transplantation (FMT)** | Promising in ICI-refractory patients, especially in melanoma; should come from responder donors | Optimal donor selection criteria, safety standardization, long-term durability of response | Lack of regulatory framework; whether defined consortia can replace donor stool |
| **Dietary Fiber** | Encourage high-fiber diet (vegetables, legumes, whole grains) for patients on ICIs | Individual variability in microbiota response to fiber | No consensus on “dose” or specific type of fiber needed for immune benefit |
| **Probiotic Supplementation** | Avoid non-targeted/generic probiotics in patients receiving ICIs | Whether personalized probiotics (strain- and patient-specific) can improve outcomes | Common use of OTC probiotics despite evidence of potential harm |
| **Microbiome Testing** | Not currently standard; potential future biomarker for ICI response prediction | Optimal methodology (16S rRNA vs shotgun sequencing); cost-effectiveness | Clinical utility and reimbursement of microbiome profiling remain debated |
| **Vidutolimod + Nivolumab** | Effective dual-immunotherapy option in some cancers when microbiome is favorable | Patient selection based on microbiome status not validated in clinical practice | Whether to co-develop this combo with microbiome-guided stratification |
| **SPEED Model and Predictive Tools** | Early data support use in rectal cancer to predict pCR with ICI | External validation and integration into routine oncology workflows | Use of AI-based microbiome tools before standard clinical validation |

| | | | |
|---|---|---|---|
| **Timing and Integration of Interventions** | Consider timing dietary interventions or FMT before ICI start | Lack of guidance on how to sequence microbiome-targeting therapies with ICIs | No standard timing or harmonization across trials and practices |
| **Microbiome and Toxicity** | Emerging data link dysbiosis with higher ICI-related adverse events | No consensus on how to mitigate toxicity via microbiome modulation | Whether to preemptively modulate microbiota to reduce immune-related AEs |

**Supplementary File S1. References of included studies.**

## References


1. Rosario, S.R.; Long, M.D.; Chilakapati, S.; Gomez, E.C.; Battaglia, S.; Singh, P.K.; Wang, J.; Wang, K.; Attwood, K.; Hess, S.M.; et al. Integrative multi-omics analysis uncovers tumor-immune-gut axis influencing immunotherapy outcomes in ovarian cancer. Nat. Commun. 2024, 15, 10609. https://doi.org/10.1038/s41467-024-54565-8.
2. Ma, J.; Sun, S.; Cheng, X.; Meng, C.; Zhao, H.; Fu, W.; Gao, Y.; Ma, L.; Yang, Z.; Yao, H.; et al. Unraveling the role of gut microbiome in predicting adverse events in neoadjuvant therapy for rectal cancer. Hum. Vaccin. Immunother. 2024, 20, 2430087. https://doi.org/10.1080/21645515.2024.2430087.
3. Ullern, A.; Holm, K.; Røssevold, A.H.; Andresen, N.K.; Bang, C.; Lingjærde, O.C.; Naume, B.; Hov, J.R.; Kyte, J.A. Gut microbiota diversity is prognostic and associated with benefit from chemo-immunotherapy in metastatic triple-negative breast cancer. Mol. Oncol. 2025, 19, 1229–1243. https://doi.org/10.1002/1878-0261.13760.
4. Sitthideatphaiboon, P.; Somlaw, N.; Zungsontiporn, N.; Ouwongprayoon, P.; Sukswai, N.; Korphaisarn, K.; Poungvarin, N.; Aporntewan, C.; Hirankarn, N.; Vinayanuwattikun, C.; et al. Dietary pattern and the corresponding gut microbiome in response to immunotherapy in Thai patients with advanced non-small cell lung cancer (NSCLC). Sci. Rep. 2024, 14, 27791. https://doi.org/10.1038/s41598-024-79339-6.
5. Zhang, M.; Bzura, A.; Baitei, E.Y.; Zhou, Z.; Spicer, J.B.; Poile, C.; Rogel, J.; Branson, A.; King, A.; Barber, S.; et al. A gut microbiota rheostat forecasts responsiveness to PD-L1 and VEGF blockade in mesothelioma. Nat. Commun. 2024, 15, 7187. https://doi.org/10.1038/s41467-024-49842-5.
6. Nakamoto, S.; Kajiwara, Y.; Taniguchi, K.; Hida, A.I.; Miyoshi, Y.; Kin, T.; Yamamoto, M.; Takabatake, D.; Kubo, S.; Hikino, H.; et al. Baseline gut microbiota as a predictive marker for the efficacy of neoadjuvant chemotherapy in patients with early breast cancer. Breast Cancer Res. Treat. 2024, 208, 67–77. https://doi.org/10.1007/s10549-024-07395-7.
7. Bukavina, L.; Ginwala, R.; Eltoukhi, M.; Sindhani, M.; Prunty, M.; Geynisman, D.M.; Ghatalia, P.; Valentine, H.; Calaway, A.; Correa, A.F.; et al. Role of gut microbiome in neoadjuvant chemotherapy response in urothelial carcinoma: A multi-institutional prospective cohort evaluation. Cancer Res. Commun. 2024, 4, 1505–1516. https://doi.org/10.1158/2767-9764.CRC-23-0479.
8. Glitza, I.C.; Seo, Y.D.; Spencer, C.N.; Wortman, J.R.; Burton, E.M.; Alayli, F.A.; Loo, C.P.; Gautam, S.; Damania, A.; Densmore, J.; et al. Randomized placebo-controlled, biomarker-stratified phase Ib microbiome modulation in melanoma: Impact of antibiotic preconditioning. Cancer Discov. 2024, 14, 1161–1175. https://doi.org/10.1158/2159-8290.CD-24-0066.
9. Routy, B.; Lenehan, J.G.; Miller, W.H. Jr.; Jamal, R.; Messaoudene, M.; Daisley, B.A.; Hes, C.; Al, K.F.; Martinez-Gili, L.; Punčochář, M.; et al. Fecal microbiota transplantation plus anti-PD-1 immunotherapy in advanced melanoma: A phase I trial. Nat. Med. 2023, 29, 2121–2132. https://doi.org/10.1038/s41591-023-02453-x.

10. Amaria, R.N.; Reddy, S.M.; Tawbi, H.A.; Davies, M.A.; Ross, M.I.; Glitza, I.C.; Cormier, J.N.; Lewis, C.; Hwu, W.-J.; Hanna, E.; et al. Neoadjuvant immune checkpoint blockade in high-risk resectable melanoma. Nat. Med. 2018, 24, 1649–1654. https://doi.org/10.1038/s41591-018-0197-1.
11. Kim, Y.; Kim, G.; Kim, S.; Cho, B.; Kim, S.-Y.; Do, E.-J.; Bae, D.-J.; Kim, S.; Kweon, M.-N.; Song, J.S.; et al. Fecal microbiota transplantation improves anti-PD-1 inhibitor efficacy in unresectable or metastatic solid cancers refractory to anti-PD-1 inhibitor. Cell Host Microbe. 2024, 32, 1380–1393.e9. https://doi.org/10.1016/j.chom.2024.06.010.
12. Cascone, T.; Leung, C.H.; Weissferdt, A.; Pataer, A.; Carter, B.W.; Godoy, M.C.B.; Feldman, H.; William, W.N., Jr.; Xi, Y.; Basu, S.; et al. Neoadjuvant chemotherapy plus nivolumab with or without ipilimumab in operable non-small cell lung cancer: The phase 2 NEOSTAR trial. Nat. Med. 2023, 29, 593–604. https://doi.org/10.1038/s41591-022-02189-0.
13. Olson, D.J.; Eroglu, Z.; Brockstein, B.; Poklepovic, A.S.; Bajaj, M.; Babu, S.; Hallmeyer, S.; Velasco, M.; Lutzky, J.; Higgs, E.; et al. Pembrolizumab plus ipilimumab following anti-PD-1/L1 failure in melanoma. J. Clin. Oncol. 2021, 39, 2647–2655. https://doi.org/10.1200/JCO.21.00079.
14. Glitza Oliva, I.C.; Ferguson, S.D.; Bassett, R., Jr.; Foster, A.P.; John, I.; Hennegan, T.D.; Rohlfs, M.; Richard, J.; Iqbal, M.; Dett, T.; et al. Concurrent intrathecal and intravenous nivolumab in leptomeningeal disease: Phase 1 trial interim results. Nat. Med. 2023, 29, 898–905. https://doi.org/10.1038/s41591-022-02170-x.
15. Weber, J.S.; Carlino, M.S.; Khattak, A.; Meniawy, T.; Ansstas, G.; Taylor, M.H.; Kim, K.B.; McKean, M.; Long, G.V.; Sullivan, R.J.; et al. Individualised neoantigen therapy mRNA-4157 (V940) plus pembrolizumab versus pembrolizumab monotherapy in resected melanoma (KEYNOTE-942): A randomized phase 2b study. Lancet 2024, 403, 632–644. https://doi.org/10.1016/S0140-673602268-7.
16. Li, H.; Zandberg, D.P.; Kulkarni, A.; Chiosea, S.I.; Santos, P.M.; Isett, B.R.; Joy, M.; Sica, G.L.; Contrera, K.J.; Tatsuoka, C.M.; et al. Distinct CD8(+) T cell dynamics associate with response to neoadjuvant cancer immunotherapies. Cancer Cell. 2025, 43, 757–775.e8. https://doi.org/10.1016/j.ccell.2025.02.026.
17. Davar, D.; Morrison, R.M.; Dzutsev, A.K.; Karunamurthy, A.; Chauvin, J.-M.; Amatore, F.; Deutsch, J.S.; Neves, R.X.D.; Rodrigues, R.R.; McCulloch, J.A.; et al. Neoadjuvant vidutolimod and nivolumab in high-risk resectable melanoma: A prospective phase II trial. Cancer Cell. 2024, 42, 1898–1918.e12. https://doi.org/10.1016/j.ccell.2024.10.007.
18. Wang, Y., Huang, S., Feng, X., Xu, W., Luo, R., Zhu, Z., Zeng, Q., & He, Z. (2023). Advances in efficacy prediction and monitoring of neoadjuvant immunotherapy for non-small cell lung cancer. *Frontiers in oncology*, *13*, 1145128. https://doi.org/10.3389/fonc.2023.1145128
19. Derosa, L.; Hellmann, M.D.; Spaziano, M.; Halpenny, D.; Fidelle, M.; Rizvi, H.; Long, N.; Plodkowski, A.J.; Arbour, K.C.; Chaft, J.E.; et al. Negative association of antibiotics on clinical activity of immune checkpoint inhibitors in patients with advanced renal cell and non–small-cell lung cancer. Ann. Oncol. 2018, 29, 1437–1444. https://doi.org/10.1093/annonc/mdy103.

20. Elkrief, A.; El Raichani, L.; Richard, C.; Messaoudene, M.; Belkaid, W.; Malo, J.; Belanger, K.; Miller, W.; Jamal, R.; Letarte, N.; et al. Antibiotics are associated with decreased progression-free survival of advanced melanoma patients treated with immune checkpoint inhibitors. Oncoimmunology 2019, 8, e1568812. https://doi.org/10.1080/2162402X.2019.1568812.
21. Andrews, M.C.; Duong, C.P.M.; Gopalakrishnan, V.; Iebba, V.; Chen, W.-S.; Derosa, L.; Khan, M.A.W.; Cogdill, A.P.; White, M.G.; Wong, M.C.; et al. Gut microbiota signatures are associated with toxicity to combined CTLA-4 and PD-1 blockade. Nat. Med. 2021, 27, 1432–1441. https://doi.org/10.1038/s41591-021-01406-6.
22. Jin, Y.; Dong, H.; Xia, L.; Yang, Y.; Zhu, Y.; Shen, Y.; Zheng, H.; Yao, C.; Wang, Y.; Lu, S. The diversity of gut microbiome is associated with favorable responses to anti–PD-1 immunotherapy in Chinese patients with NSCLC. J. Thorac. Oncol. 2019, 14, 1378–1389. https://doi.org/10.1016/j.jtho.2019.04.007.
23. Peng, Z.; Cheng, S.; Kou, Y.; Wang, Z.; Jin, R.; Hu, H.; Zhang, X.; Gong, J.-F.; Li, J.; Lu, M.; et al. The gut microbiome is associated with clinical response to anti–PD-1/PD-L1 immunotherapy in gastrointestinal cancer. Cancer Immunol. Res. 2020, 8, 1251–1261. https://doi.org/10.1158/2326-6066.CIR-20-0303.
24. Coutzac, C.; Jouniaux, J.M.; Paci, A.; Tang, Y.; Ji, J.; Wang, H.; Karim, R.; Rosas, C.; Huang, Y.; Zhai, Y. Antibiotics are associated with attenuated efficacy of immune checkpoint blockers in patients with advanced renal cell and non–small-cell lung cancer. Ann. Oncol. 2019, 30, 651–659. https://doi.org/10.1093/annonc/mdz027.
25. Hakozaki, T.; Richard, C.; Elkrief, A.; Hosomi, Y.; Benlaïfaoui, M.; Mimpen, I.; Terrisse, S.; Derosa, L.; Zitvogel, L.; Routy, B.; et al. The gut microbiome associates with immune checkpoint inhibition outcomes in patients with advanced NSCLC. Cancer Immunol. Res. 2020, 8, 1243–1250. https://doi.org/10.1158/2326-6066.CIR-19-1064.
26. Gazzaniga FS, Kasper DL. The gut microbiome and cancer response to immune checkpoint inhibitors. J Clin Invest. 2025;135(1):e178897. doi:10.1172/JCI178897. PubMed PMID: 39895632
27. Botticelli, A.; Zizzari, I.G.; Mazzuca, F.; Ascierto, P.A.; Putignani, L.; Marchetti, L.; Napoletano, C.; Nuti, M.; Marchetti, P. Cross-talk between microbiota and immune fitness to steer and control response to anti–PD-1/PD-L1 treatment. J. Exp. Clin. Cancer Res. 2019, 38, 264. https://doi.org/10.1186/s13046-019-1258-z.
28. Raziq, M. F., Manzoor, H., & Kayani, M. U. R. (2025). Non-small Cell Lung Cancer, Immunotherapy and the Influence of Gut Microbiome. *Current microbiology*, *82*(9), 419. https://doi.org/10.1007/s00284-025-04408-6
29. Wang, Y.; Wiesnoski, D.H.; Helmink, B.A.; Gopalakrishnan, V.; Choi, K.; DuPont, H.L.; Jiang, Z.-D.; Abu-Sbeih, H.; Sanchez, C.A.; Chang, C.-C.; et al. Fecal microbiota transplantation for refractory immune checkpoint inhibitor–associated colitis. Nat. Med. 2018, 24, 1804–1808. https://doi.org/10.1038/s41591-018-0238-2.
30. Kaderbhai, C.; Richard, C.; Fumet, J.D.; Aarnink, A.; Foucher, P.; Coudert, B.; Favier, L.; Lagrange, A.; Limagne, E.; Boidot, R.; et al. Antibiotic use does not appear to influence response to immune checkpoint blockade. Anticancer. Res. 2017, 37, 3195–3200. https://doi.org/10.21873/anticanres.11637.

31. Grenda A, Iwan E, Kuźnar-Kamińska B, Bomba A, Bielińska K, Krawczyk P, Chmielewska I, Frąk M, Szczyrek M, Rolska-Kopińska A, et al. Gut microbial predictors of first-line immunotherapy efficacy in advanced NSCLC patients. Sci Rep. 2025;15:6139. doi:10.1038/s41598-025-89406-1. PubMed PMID: 39979394.
32. Xia, L., Zhu, X., Wang, Y., & Lu, S. (2024). The gut microbiota improves the efficacy of immune-checkpoint inhibitor immunotherapy against tumors: From association to cause and effect. *Cancer letters*, *598*, 217123. https://doi.org/10.1016/j.canlet.2024.217123
33. Petrelli F, Iaculli A, Signorelli D, Ghidini A, Dottorini L, Perego G, Cabiddu M, Borgonovo K, Parati MC, Ghilardi M, et al. Survival of patients treated with antibiotics and immunotherapy for cancer: a systematic review and meta-analysis. J Clin Med. 2020;9(5):1458. doi:10.3390/jcm9051458. PubMed PMID: 32414103.
34. Nomura, M.; Nagatomo, R.; Doi, K.; Shimizu, J.; Baba, K.; Saito, T.; Matsumoto, S.; Inoue, K.; Muto, M. Association of short-chain fatty acids in the gut with response to nivolumab in advanced non–small cell lung cancer. Transl. Lung. Cancer Res. 2020, 9, 1900–1909. https://doi.org/10.21037/tlcr-20-412.
35. Li, Y.; Tinoco, R.; Elmén, L.; Segota, I.; Xian, Y.; Fujita, Y.; Sahu, A.; Zarecki, R.; Marie, K.; Feng, Y.; et al. Gut microbiota dependent anti-tumor immunity restricts melanoma growth in Rnf5−/− mice. Nat. Commun. 2019, 10, 1492. https://doi.org/10.1038/s41467-019-09525-y.
36. Vétizou, M.; Pitt, J.M.; Daillère, R.; Lepage, P.; Waldschmitt, N.; Flament, C.; Rusakiewicz, S.; Routy, B.; Roberti, M.P.; Duong, C.P.M.; et al. Anticancer immunotherapy by CTLA-4 blockade relies on the gut microbiota. Science 2015, 350, 1079–1084. https://doi.org/10.1126/science.aad1329.
37. Sivan, A.; Corrales, L.; Hubert, N.; Williams, J.B.; Aquino-Michaels, K.; Earley, Z.M.; Benyamin, F.W.; Lei, Y.M.; Jabri, B.; Alegre, M.-L.; et al. Commensal Bifidobacterium promotes antitumor immunity and facilitates anti–PD-L1 efficacy. Science 2015, 350, 1084–1089. https://doi.org/10.1126/science.aac4255.
38. Geller, L.T.; Barzily-Rokni, M.; Danino, T.; Jonas, O.H.; Shental, N.; Nejman, D.; Gavert, N.; Zwang, Y.; Cooper, Z.A.; Shee, K.; et al. Potential role of intratumor bacteria in mediating tumor resistance to the chemotherapeutic drug gemcitabine. Science 2017, 357, 1156–1160. https://doi.org/10.1126/science.aah5043.
39. Ma, C.; Han, M.; Heinrich, B.; Fu, Q.; Zhang, Q.; Sandhu, M.; Agdashian, D.; Terabe, M.; Berzofsky, J.A.; Fako, V.; et al. Gut microbiome–mediated bile acid metabolism regulates liver cancer via NKT cells. Science 2018, 360, eaan5931. https://doi.org/10.1126/science.aan5931.
40. Zitvogel, L.; Ma, Y.; Raoult, D.; Kroemer, G.; Gajewski, T.F. The microbiome in cancer immunotherapy: Diagnostic tools and therapeutic strategies. Science 2018, 359, 1366–1370. https://doi.org/10.1126/science.aar6918.
41. Gopalakrishnan, V.; Helmink, B.A.; Spencer, C.N.; Reuben, A.; Wargo, J.A. The influence of the gut microbiome on cancer, immunity, and cancer immunotherapy. Cancer Cell. 2018, 33, 570–580. https://doi.org/10.1016/j.ccell.2018.03.015.
42. Suez, J.; Zmora, N.; Zilberman-Schapira, G.; Mor, U.; Dori-Bachash, M.; Bashiardes, S.; Zur, M.; Regev-Lehavi, D.; Brik, R.B.-Z.; Federici, S.; et al. Post-antibiotic gut mucosal microbiome reconstitution is impaired by probiotics and improved by autologous FMT. Cell 2018, 174, 1406–1423.e16. https://doi.org/10.1016/j.cell.2018.08.047.

43. Tanoue, T.; Atarashi, K.; Honda, K. Development and maintenance of intestinal regulatory T cells. Nat. Rev. Immunol. 2016, 16, 295–309. https://doi.org/10.1038/nri.2016.36.
44. Rosshart, S.P.; Herz, J.; Vassallo, B.G.; Hunter, A.; Wall, M.K.; Badger, J.H.; McCulloch, J.A.; Anastasakis, D.G.; Sarshad, A.A.; Leonardi, I.; et al. Laboratory mice born to wild mice have natural microbiota and model human immune responses. Science 2019, 365, eaaw4361. https://doi.org/10.1126/science.aaw4361.
45. Baruch, E.N.; Ben-Betzalel, G.; Elinav, E.; et al. Fecal microbiota transplantation for refractory melanoma patients: Mechanisms and predictive biomarkers. Cell Host Microbe. 2021, 29, 844–853.e5. https://doi.org/10.1016/j.chom.2021.03.020.
46. Ma, W.; Mao, Q.; Xia, W.; Dong, G.; Yu, C.; Jiang, F. Gut microbiota shapes the efficiency of cancer therapy. Front. Microbiol. 2019, 10, 1050. https://doi.org/10.3389/fmicb.2019.01050.
47. Liu W, Wang Y, Luo J, Liu M, Luo Z. Pleiotropic Effects of Metformin on the Antitumor Efficiency of Immune Checkpoint Inhibitors. *Front Immunol*. 2021;11:586760. Published 2021 Feb 2. doi:10.3389/fimmu.2020.586760
48. Cheng WY, Wu CY, Yu J. The role of gut microbiota in cancer treatment: friend or foe? Gut. 2020;69(10):1867-1876. doi:10.1136/gutjnl-2020-321153. PubMed PMID: 32759302.
49. Iida, N.; Dzutsev, A.; Stewart, C.A.; Smith, L.; Bouladoux, N.; Weingarten, R.A.; Molina, D.A.; Salcedo, R.; Back, T.; Cramer, S.; et al. Commensal bacteria control cancer response to therapy by modulating the tumor microenvironment. Science 2013, 342, 967–970. https://doi.org/10.1126/science.1240527.
50. Peled, J.U.; Gomes, A.L.C.; Devlin, S.M.; Littmann, E.R.; Taur, Y.; Sung, A.D.; Weber, D.; Hashimoto, D.; Slingerland, A.E.; Slingerland, J.B.; et al. Microbiota as predictor of mortality in allogeneic hematopoietic-cell transplantation. N. Engl. J. Med. 2020, 382, 822–834. https://doi.org/10.1056/NEJMoa1900623.
51. Oh B, Boyle F, Pavlakis N, Clarke S, Eade T, Hruby G, Lamoury G, Carroll S, Morgia M, Kneebone A, et al. The gut microbiome and cancer immunotherapy. Cancers (Basel). 2021;13(19):4824. doi:10.3390/cancers13194824. PubMed PMID: 34638308.
52. Nejman, D.; Livyatan, I.; Fuks, G.; Gavert, N.; Zwang, Y.; Geller, L.T.; Rotter-Maskowitz, A.; Weiser, R.; Mallel, G.; Gigi, E.; Meltser, A.; et al. The human tumor microbiome is composed of tumor type-specific intracellular bacteria. Science 2020, 368, 973–980. https://doi.org/10.1126/science.aay9189.
53. Kostic, A.D.; Chun, E.; Robertson, L.; Glickman, J.N.; Gallini, C.A.; Michaud, M.; Clancy, T.E.; Chung, D.C.; Lochhead, P.; Hold, G.L.; et al. Fusobacterium nucleatum potentiates intestinal tumorigenesis and modulates the tumor-immune microenvironment. Cell Host Microbe. 2013, 14, 207–215. https://doi.org/10.1016/j.chom.2013.07.007.
54. Ghosh, T.S.; Shanahan, F.; O'Toole, P.W. The gut microbiome as a modulator of healthy ageing. Nat. Rev. Gastroenterol. Hepatol. 2022, 19, 565–584. https://doi.org/10.1038/s41575-022-00635-z.
55. Sholl, L.M.; Hirsch, F.R.; Hwang, D.; Botling, J.; Lopez-Rios, F.; Bubendorf, L.; Mino-Kenudson, M.; Roden, A.C.; Beasley, M.B.; Borczuk, A.; et al. The promises and challenges of tumor mutation burden as an immunotherapy biomarker: A perspective from the International Association for the Study of Lung Cancer Pathology Committee. J. Thorac. Oncol. 2020, 15, 1409–1424. https://doi.org/10.1016/j.jtho.2020.05.019.

56. Sepich-Poore, G.D.; Zitvogel, L.; Straussman, R.; Hasty, J.; Wargo, J.A.; Knight, R. The microbiome and human cancer. Science 2021, 371, eabc4552. https://doi.org/10.1126/science.abc4552.
57. Choi, Y.; Lichterman, J.N.; Coughlin, L.A.; Poulides, N.; Li, W.; Del Valle, P.; Palmer, S.N.; Gan, S.; Kim, J.; Zhan, X.; et al. Immune checkpoint blockade induces gut microbiota translocation that augments extraintestinal antitumor immunity. Sci. Immunol. 2023, 8, eabo2003. https://doi.org/10.1126/sciimmunol.abo2003.
58. Cass, S.; White, M.G. The Influence of the Microbiome on Metastatic Colorectal Cancer. Clin. Colon Rectal Surg. 2023, 36, 112–119. https://doi.org/10.1055/s-0043-1760864.
59. Xu, H.; Cao, C.; Ren, Y.; Weng, S.; Weng, S.; Liu, L.; Guo, C.; Wang, L.; Han, X.; Ren, J.; Liu, Z. Antitumor effects of fecal microbiota transplantation: Implications for microbiome modulation in cancer treatment. Front. Immunol. 2022, 13, 949490. https://doi.org/10.3389/fimmu.2022.949490.
60. Michikawa, C.; Gopalakrishnan, V.; Harrandah, A.M.; Karpinets, T.V.; Garg, R.R.; Chu, R.A.; Park, Y.P.; Chukkapallia, S.S.; Yadlapalli, N.; Erikson-Carter, K.C.; et al. Fusobacterium is enriched in oral cancer and promotes induction of programmed death-ligand 1 (PD-L1). Neoplasia 2022, 31, 100813. https://doi.org/10.1016/j.neo.2022.100813.
61. Lu, Y.; Yuan, X.; Wang, M.; He, Z.; Li, H.; Wang, J.; Li, Q. Gut microbiota influence immunotherapy responses: mechanisms and therapeutic strategies. J. Hematol. Oncol. 2022, 15, 47. https://doi.org/10.1186/s13045-022-01273-9.
62. Silva, M.; Brunner, V.; Tschurtschenthaler, M. Microbiota and Colorectal Cancer: From Gut to Bedside. Front. Pharmacol. 2021, 12, 760280. https://doi.org/10.3389/fphar.2021.760280.
63. Gopalakrishnan, V.; Weiner, B.; Ford, C.B.; Sellman, B.R.; Hammond, S.A.; Freeman, D.J.; Dennis, P.; Soria, J.-C.; Wortman, J.R.; Henn, M.R. Intervention strategies for microbial therapeutics in cancer immunotherapy. Immunooncol. Technol. 2020, 6, 9–17. https://doi.org/10.1016/j.iotech.2020.05.001.
64. Gunjur, A., Shao, Y., Rozday, T., Klein, O., Mu, A., Haak, B. W., Markman, B., Kee, D., Carlino, M. S., Underhill, C., Frentzas, S., Michael, M., Gao, B., Palmer, J., Cebon, J., Behren, A., Adams, D. J., & Lawley, T. D. (2024). A gut microbial signature for combination immune checkpoint blockade across cancer types. *Nature medicine*, *30*(3), 797–809. https://doi.org/10.1038/s41591-024-02823-z
65. Naqash, A. R., Kihn-Alarcón, A. J., Stavraka, C., Kerrigan, K., Maleki Vareki, S., Pinato, D. J., & Puri, S. (2021). The role of gut microbiome in modulating response to immune checkpoint inhibitor therapy in cancer. *Annals of translational medicine*, *9*(12), 1034. https://doi.org/10.21037/atm-20-6427
66. Chang CW, Lee HC, Li LH, Chang YH, Wu MS, Lin JT, Wang HP, Chen CC. Fecal microbiota transplantation alleviates immune checkpoint inhibitor-induced colitis. Clin Transl Gastroenterol. 2020;11(2):e00134. doi:10.14309/ctg.0000000000000134
67. Wang F, Yin Q, Chen L, Davis MM, Zhang C. Gut microbiome composition modulates the antitumor effect of PD-1 blockade in murine models. Cell Rep. 2018;23(12):3891-3903. doi:10.1016/j.celrep.2018.05.093
68. Chaput N, Lepage P, Coutzac C, Soularue E, Le Roux K, Monot C, Boselli L, Routier E, Cassard L, Collins M, et al. Baseline gut microbiota predicts clinical response and colitis in metastatic melanoma patients treated with ipilimumab. Ann Oncol. 2017;28(6):1368-1379. doi:10.1093/annonc/mdx108

69. Dubin K, Callahan MK, Ren B, Khanin R, Viale A, Ling L, No D, Gobourne A, Littmann E, Huttenhower C, et al. Intestinal microbiome analyses identify melanoma patients at risk for checkpoint-blockade-induced colitis. Nat Commun. 2016;7:10391. doi:10.1038/ncomms10391
70. Zheng Y, Wang T, Tu X, Huang Y, Zhang H, Tan D, Jiang W, Cai S, Zhao P, Song R, et al. Gut microbiome affects the response to anti–PD-1 immunotherapy in patients with hepatocellular carcinoma. J Immunother Cancer. 2019;7(1):193. doi:10.1186/s40425-019-0649-3
71. Peters BA, Wilson M, Moran U, Pavlick A, Izsak A, Wechter T, Weber JS, Osman I, Ahn J. Relating the gut microbiome and outcomes in patients with advanced melanoma treated with immune checkpoint inhibitors. J Natl Cancer Inst. 2019;111(6):616-622. doi:10.1093/jnci/djy185
72. Lee KA, Thomas AM, Bolte LA, Björk JR, de Ruijter LK, Armanini F, de Vos WM, Reijnders D, Adriaans D, Dekker E, et al. Cross-cohort gut microbiome associations with immune checkpoint inhibitor response in advanced melanoma. Nat Med. 2022;28(8):1620-1631. doi:10.1038/s41591-022-01964-3
73. McQuade JL, Daniel CR, Helmink BA, Wargo JA. Modulating the microbiome to improve therapeutic response in cancer. Lancet Oncol. 2019;20(2):e77-e91. doi:10.1016/S1470-2045(18)30952-5
74. Routy, B.; le Chatelier, E.; DeRosa, L.; Duong, C.P.M.; Alou, M.T.; Daillère, R.; Fluckiger, A.; Messaoudene, M.; Rauber, C.; Roberti, M.P.; et al. Gut microbiome influences efficacy of PD-1–based immunotherapy against epithelial tumors. *Science* 2018, 359, 91–97. https://doi.org/10.1126/science.aan3706.
75. Gopalakrishnan, V.; Spencer, C.N.; Nezi, L.; Reuben, A.; Andrews, M.C.; Karpinets, T.V.; Prieto, P.A.; Vicente, D.; Hoffman, K.; Wei, S.C.; et al. Gut microbiome modulates response to anti–PD-1 immunotherapy in melanoma patients. *Science* 2018, 359, 97–103. https://doi.org/10.1126/science.aan4236.
76. Matson, V.; Fessler, J.; Bao, R.; Chongsuwat, T.; Zha, Y.; Alegre, M.-L.; Luke, J.J.; Gajewski, T.F. The commensal microbiome is associated with anti–PD-1 efficacy in metastatic melanoma patients. *Science* 2018, 359, 104–108. https://doi.org/10.1126/science.aao3290.
77. Frankel, A.E.; Coughlin, L.A.; Kim, J.; Froehlich, T.W.; Xie, Y.; Frenkel, E.P.; Koh, A.Y. Metagenomic shotgun sequencing and unbiased metabolomic profiling identify specific human gut microbiota and metabolites associated with immune checkpoint therapy efficacy in melanoma. *J. Immunother. Cancer* 2017, 5, 18. https://doi.org/10.1016/j.neo.2017.08.004.
78. Davar, D.; Dzutsev, A.K.; McCulloch, J.A.; Rodrigues, R.R.; Chauvin, J.-M.; Morrison, R.M.; Deblasio, R.N.; Menna, C.; Ding, Q.; Pagliano, O.; et al. Fecal microbiota transplant overcomes resistance to anti–PD-1 therapy in melanoma patients. *Science* 2021, 371, 595–602. https://doi.org/10.1126/science.abf3363.
79. Baruch, E.N.; Youngster, I.; Ben-Betzalel, G.; Ortenberg, R.; Lahat, A.; Katz, L.; Adler, K.; Dick-Necula, D.; Raskin, S.; Bloch, N.; et al. Fecal microbiota transplant promotes response in immunotherapy-refractory melanoma patients. *Science* 2021, 371, 602–609. https://doi.org/10.1126/science.abb5920.

80. Yang, Z.; Ma, J.; Han, J.; Li, A.; Liu, G.; Sun, Y.; Zheng, J.; Zhang, J.; Chen, G.; Xu, R.; et al. Gut microbiome model predicts response to neoadjuvant immunotherapy plus chemoradiotherapy in rectal cancer. Med 2024, 5, 1293–1306.e4. https://doi.org/10.1016/j.medj.2024.07.002. PMID: 39047732.
81. Choueiri, T.K.; Albiges, L.; Haanen, J.B.A.G.; Larkin, J.M.; Uemura, M.; Pal, S.K.; Gravis, G.; Campbell, M.T.; Penkov, K.; Lee, J.-L.; et al. The gut microbiome (GM) and probiotics influence response to immune checkpoint blockade (ICB) in melanoma. J. Clin. Oncol. 2019, 37 (Suppl. 15), 101. https://doi.org/10.1200/JCO.2019.37.15_suppl.101.
82. Ribas, A.; Medina, T.; Kummar, S.; Amin, A.; Kalbasi, A.; Drabick, J.J.; Barve, M.; Daniels, G.A.; Wong, D.J.; Schmidt, E.V.; et al. SD-101 in combination with pembrolizumab in advanced melanoma: Results of a phase 1b, multicenter study. Cancer Discov. 2018, 8, 1250–1257. https://doi.org/10.1158/2159-8290.CD-18-0280.
83. Derosa, L.; Routy, B.; Fidelle, M.; Iebba, V.; Alla, L.; Pasolli, E.; Segata, N.; Desnoyer, A.; Pietrantonio, F.; Ferrere, G.; et al. Gut bacteria composition drives primary resistance to cancer immunotherapy in renal cell carcinoma patients. Eur. Urol. 2020, 78, 195–206. https://doi.org/10.1016/j.eururo.2020.04.044.
84. Pinato, D.J.; Howlett, S.; Ottaviani, D.; Urus, H.; Patel, A.; Mineo, T.; Brock, C.; Power, D.; Hatcher, O.; Falconer, A.; et al. Association of prior antibiotic treatment with survival and response to immune checkpoint inhibitor therapy in patients with cancer. JAMA Oncol. 2019, 5, 1774–1778. https://doi.org/10.1001/jamaoncol.2019.2785.
85. Wilson, B.E.; Routy, B.; Nagrial, A.; Chin, V.T. The effect of antibiotics on clinical outcomes in immune-checkpoint blockade: A systematic review and meta-analysis of observational studies. Cancer Immunol. Immunother. 2020, 69, 343–354. https://doi.org/10.1007/s00262-019-02453-2.
86. Giampazolias, E.; da Costa, M.P.; Lam, K.C.; Lim, K.H.J.; Cardoso, A.; Piot, C.; Chakravarty, P.; Blasche, S.; Patel, S.; Biram, A.; et al. Vitamin D regulates microbiome-dependent cancer immunity. Science 2024, 384, 428–437. https://doi.org/10.1126/science.adh7954.
87. Elkrief, A.; Derosa, L.; Kroemer, G.; Zitvogel, L.; Routy, B. The negative impact of antibiotics on outcomes in cancer patients treated with immunotherapy: A new independent prognostic factor? *Ann. Oncol.* 2019, 30, 1572–1579. https://doi.org/10.1093/annonc/mdz206.
88. Dizman, N.; Meza, L.; Bergerot, P.; Alcantara, M.; Dorff, T.; Lyou, Y.; Frankel, P.; Cui, Y.; Mira, V.; Llamas, M.; et al. Nivolumab plus ipilimumab with or without live bacterial supplementation in metastatic renal cell carcinoma: A randomized phase 1 trial. *Nat. Med.* 2022, 28, 704–712. https://doi.org/10.1038/s41591-022-01694-6.
89. Wastyk, H.C.; Fragiadakis, G.K.; Perelman, D.; Dahan, D.; Merrill, B.D.; Yu, F.B.; Topf, M.; Gonzalez, C.G.; Van Treuren, W.; Han, S.; et al. Gut-microbiota-targeted diets modulate human immune status. *Cell* 2021, 184, 4137–4153.e14. https://doi.org/10.1016/j.cell.2021.06.019.
90. Spencer, C.N.; McQuade, J.L.; Gopalakrishnan, V.; McCulloch, J.A.; Vetizou, M.; Cogdill, A.P.; Khan, A.W.; Zhang, X.; White, M.G.; Peterson, C.B.; et al. Dietary fiber and probiotics influence the gut microbiome and melanoma immunotherapy response. *Science* 2021, 374, 1632–1640. https://doi.org/10.1126/science.aaz7015.

91. Tanoue, T.; Morita, S.; Plichta, D.R.; Skelly, A.N.; Suda, W.; Sugiura, Y.; Narushima, S.; Vlamakis, H.; Motoo, I.; Sugita, K.; et al. A defined commensal consortium elicits CD8 T cells and anti-cancer immunity. *Nature* 2019, 565, 600–605. https://doi.org/10.1038/s41586-019-0878-z.
92. Helmink, B.A.; Khan, M.A.W.; Hermann, A.; Gopalakrishnan, V.; Wargo, J.A. The microbiome, cancer, and cancer therapy. *Nat. Med.* 2019, 25, 377–388. https://doi.org/10.1038/s41591-019-0377-7.
93. McCulloch, J. A., Davar, D., Rodrigues, R. R., Badger, J. H., Fang, J. R., Cole, A. M., Balaji, A. K., Vetizou, M., Prescott, S. M., Fernandes, M. R. Intestinal microbiota signatures of clinical response and immune-related adverse events in melanoma patients treated with anti-PD-1. Nature medicine, 2022, 28, 545–556. https://doi.org/10.1038/s41591-022-01698-2.
94. Petersen, C.; Round, J.L. Defining dysbiosis and its influence on host immunity and disease. *Cell Microbiol.* 2014, 16, 1024–1033. https://doi.org/10.1111/cmi.12308.
95. Chen, Y.; Zhao, N.; Xu, L.; Jia, X.; Liu, F.; Huang, J.; Li, X.; Wang, Y.; Lai, C.; Shen, Y.; et al. Integrative multi-omics analysis reveals the LncRNA 60967.1-PLCD4-ATRA axis as a key regulator of colorectal cancer progression and immune response. Mol. Cancer 2025, 24, 164. https://doi.org/10.1186/s12943-025-02359-x.

**Supplementary File S2. Systematic review search strategy.**

(("gut microbiome" OR "intestinal microbiota" OR "microbiota" OR "fecal microbiota transplantation" OR "FMT" OR "probiotic*" OR "prebiotic*" OR "dietary fiber")

AND ("immune checkpoint inhibitor*" OR "PD-1" OR "PD-L1" OR "CTLA-4" OR "immunotherapy")

AND (cancer OR carcinoma OR tumor OR neoplasm))

Filters: Humans, 2013–2025, Clinical Trial OR Observational Study

**Supplementary File S3. PRISMA flow diagram of included studies.**

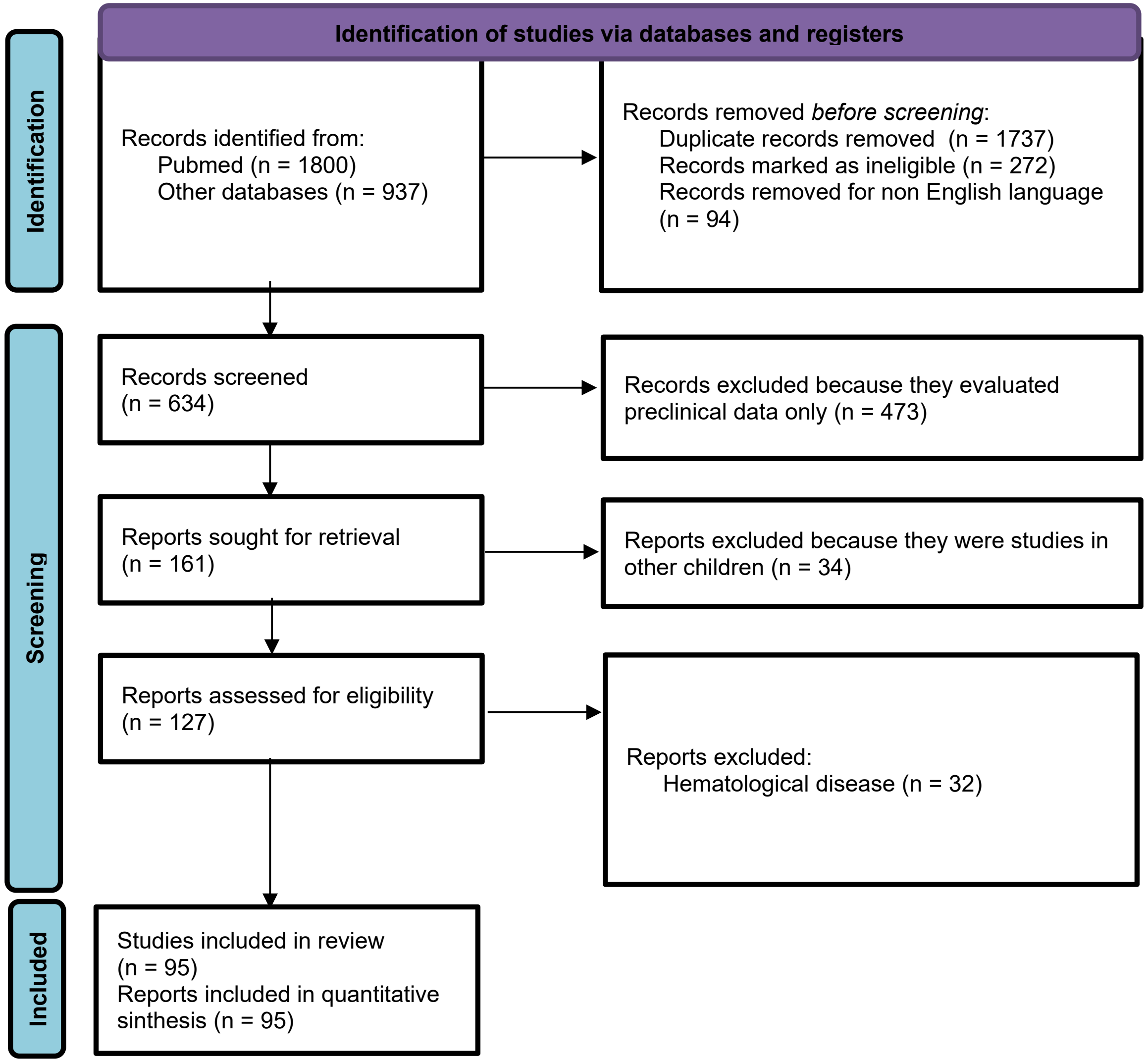

Supplement: Supplementary file 1 [file medicina-61-01595-s001.zip › medicina-3833275-supplementary.pdf]
